# Radiomics is feasible for prediction of spread through air spaces in patients with nonsmall cell lung cancer

**DOI:** 10.1038/s41598-021-93002-4

**Published:** 2021-06-29

**Authors:** Yuki Onozato, Takahiro Nakajima, Hajime Yokota, Jyunichi Morimoto, Akira Nishiyama, Takahide Toyoda, Terunaga Inage, Kazuhisa Tanaka, Yuichi Sakairi, Hidemi Suzuki, Takashi Uno, Ichiro Yoshino

**Affiliations:** 1 Departments of General Thoracic Surgery, Chiba University Graduate School of Medicine, 1-8-1, Chuo-ku, Inohana, Chiba, 260-8670 Japan; 2 Department of Diagnostic Radiology and Radiation Oncology, Chiba University Graduate School of Medicine, Chiba, Japan; 3 Department of Radiology, Chiba University Hospital, Chiba, Japan

**Keywords:** Non-small-cell lung cancer, Image processing

## Abstract

Tumor spread through air spaces (STAS) in non-small-cell lung cancer (NSCLC) is known to influence a poor patient outcome, even in patients presenting with early-stage disease. However, the pre-operative diagnosis of STAS remains challenging. With the progress of radiomics-based analyses several attempts have been made to predict STAS based on radiological findings. In the present study, patients with NSCLC which is located peripherally and tumors ≤ 2 cm in size on computed tomography (CT) that were potential candidates for sublobar resection were enrolled in this study. The radiologic features of the targeted tumors on thin-section CT were extracted using the PyRadiomics v3.0 software package, and a predictive model for STAS was built using the t-test and XGBoost. Thirty-five out of 226 patients had a STAS histology. The predictive model of STAS indicated an area under the receiver-operator characteristic curve (AUC) of 0.77. There was no significant difference in the overall survival (OS) for lobectomy between the predicted-STAS (+) and (−) groups (*p* = 0.19), but an unfavorable OS for sublobar resection was indicated in the predicted-STAS (+) group (*p* < 0.01). These results suggest that radiomics with machine-learning helped to develop a favorable model of STAS (+) NSCLC, which might be useful for the proper selection of candidates who should undergo sublobar resection.

## Introduction

Kawakami et al. first reported the findings for tumor spread through air spaces (STAS) in pulmonary adenocarcinoma in 2008^1^. The 4th Edition WHO Classification of Tumor of The Lung Pleura, Thymus and Heart describe STAS as a novel invasive morphology depicted as micropapillary clusters, solid nests, or single cells beyond the edge of the tumor spreading into air spaces in the surrounding lung parenchyma^2^. STAS is often associated with aggressive subtypes of pulmonary adenocarcinoma, such as micropapillary and solid pattern, and 30–50% of pulmonary adenocarcinomas manifest STAS findings. Even in early-stage disease, positivity for STAS is regarded as a poor prognostic characteristic^3,4^. In addition to adenocarcinoma histology, STAS coexists with other histologies, such as squamous cell carcinoma, pleomorphic carcinoma and neuroendocrine carcinoma. A poor postoperative outcome is a common feature for STAS-positive non-small-cell lung cancer^5–8^.

The standard procedure for lung cancer has been lobectomy with lymph node dissection, and patients with small lung cancer (≤ 2 cm) have shown an excellent long-term survival after lobectomy^9^. However, the efficacy of sublobar resection for managing small lung cancer (≤ 2 cm) remains controversial^10,11^. In a nonrandomized confirmatory phase III trial that assessed sublobar resection for peripheral ground-glass opacity (GGO)-dominant lung cancer, such as those with a maximum tumor diameter ≤ 2 cm and a consolidation/tumor ratio (C/T ratio) ≤ 0.25 on thin-section CT (Japan Clinical Oncology Group 0804/WJOG4507L), sublobar resection was shown to offer a sufficient degree of local control and recurrence-free survival^12^. We are now awaiting the results of a randomized controlled trial to confirm the noninferiority of segmentectomy to lobectomy with regard to the prognosis according to the JCOG0802/WJOG4607L trial, although the safety of segmentectomy has already been reported (JCOG0802/WJOG4607L)^13^.

Sublobar resection has been reported to have a poor prognosis in patients with a micropapillary component of ≥ 5%, even for small lung cancers with a maximum tumor diameter of ≤ 2 cm^14^. The preoperative diagnosis of aggressive histological subtypes, including STAS, is important to determine the proper operative procedure; however, making pathological and radiological diagnoses before surgery remains difficult. Regarding radiology, a larger tumor size and greater degree of solid component were reportedly correlated with the presence of STAS^15,16^; however, no evidence has been obtained concerning correlations for small, peripheral tumors (< 2 cm), which may be candidates for limited surgery, such as segmentectomy. It was also shown to be difficult to diagnose STAS by cytology, with a reported sensitivity of 45%, which was not sufficient for a pre-surgical diagnosis^17^.

Predicting aggressive histological subtypes or STAS preoperatively by imaging using novel image analysis technologies might therefore help surgeons determine the optimal surgical treatment plan.

With the introduction of radiomics-based machine-learning analyses, several reports have provided new insight into aspects such as the diagnosis of cancer and prognosis prediction^18–20^. Recently, two groups reported that radiomics-based analyses of CT findings could predict the existence of STAS in lung adenocarcinoma^21,22^.

STAS is observed in several histologies, and its existence is known to be related to the surgical outcome. We therefore retrospectively reviewed the histology and CT findings of patients who underwent surgical resection for ≤ 2 cm lung cancers. Using preoperative thin-section CT images, we developed a radiomics-based machine-learning prediction model of STAS-positive lung cancer, without using any clinical information. We also conducted a survival analysis comparing the radiomics results and actual histological STAS results to assess the utility of the prediction model as a preoperative surgical planning tool.

## Methods

### Patient selection

We retrospectively reviewed the patients who underwent surgical resection of lung cancer between January 2013 and December 2018 at our institution. Peripheral lung tumors with the maximum axial diameter should be ≤ 2 cm that were suitable for limited surgery were included, and cases with pre-operative or frozen sections during surgery proving the presence of small-cell lung cancer were excluded from this study. Preoperative thin-section plain CT (≤ 1 mm slice thickness) performed within 2 months prior to surgery was mandatory for eligibility.

The clinical information and follow-up data were obtained from electronic medical records. The clinicopathologic features, including the age, sex, smoking history, pulmonary function, CT images, surgical procedure, pathological diagnosis following the 7th edition of TNM classification, histologic subtype following the 2015 WHO classification, and EGFR mutation status, were examined. Perioperative surgical treatment was performed following the standard thoracic surgery guidelines^23^. The standard surgical procedure for lung cancer at our institute is lobectomy with systematic lymph node dissection, but limited surgery is adopted for peripheral GGO-dominant lung cancer or patients who are compromised, such as those with comorbidities, a low pulmonary function, or an older age who are indicated for palliative care.

Routine postoperative follow-up included regular physical examinations, blood tests, chest X-ray, and chest CT. Fluorodeoxyglucose-positron emission tomography (FDG-PET) and brain magnetic resonance imaging (MRI) were performed on demand according to the physician’s decision.

### The histological diagnosis

The histological diagnostic information was obtained from official pathology reports made by board-certified pathologists. All formalin-fixed, paraffin-embedded 4-μm sections with hematoxylin–eosin staining were then re-evaluated by a pathology-trained physician (J.M.) to confirm the STAS diagnosis. We previously reported free tumor clusters as a risk factor for a poor prognosis^24^. Following the latest WHO classification^2^, STAS was defined as micropapillary clusters, solid nests, or single cells beyond the edge of the tumor extending into the air spaces in the surrounding lung parenchyma. To avoid false positives due to extraneous factors, such as artifacts induced by cutting though a tumor with a knife^25^, we developed criteria to diagnose STAS by histology. For example, haphazardly distributed fragments of a tumor cells (clusters) with sharp jagged edges on microscopy were regarded as “artifacts STAS” which meant artifactual floaters caused by the steps of making histological sections by knife. We excluded such artifacts STAS from our STAS criteria^26^. To avoid compromising the reproducibility of the STAS assessment, STAS was defined as > 3 clusters floating in an alveolar space > 3 mm away from the main tumor margin. Regarding intrinsic factors, the invasive mucinous adenocarcinoma manifest goblet or columnar tumor cells spread diffusely to the surrounding alveoli via the airway. Therefore, invasive mucinous adenocarcinoma usually shows a STAS-like growth pattern and thus it cannot be regarded as STAS in this study.

### Chest CT and tumor segmentation

All patients underwent chest CT within two months prior to surgery. Chest CT was performed in the supine position during an inspiratory breath-hold using various multidetector row scanners: an Aquilion prime (CANON MEDICAL SYSTEMS CORPORATION, Tochigi, Japan), Aquilion ONE (CANON), Alexion (CANON), Activion16 (CANON), and Aquilion64 (CANON). All CT data were resampled at 0.5 × 0.5 × 1.0 mm resolution with trilinear interpolation.

Targeted lung cancer was segmented with a semiautomated method using the 3D Slicer software program, ver. 4.10.2 (https://www.slicer.org/). Lung cancer was denoted with solid and ground-glass components. We defined solid components as those of >  − 80 Hounsfield Unit (HU) and ground-glass opacity as components of >  − 600 HU and <  − 80 HU. The volume of interest (VOI) of lung cancer was delineated by the fusion of the solid and ground-glass parts.

### Feature extraction and machine learning

Imaging features were extracted using the PyRadiomics v3.0 open-source software program (http://www.radiomics.io/pyradiomics.html^27^. Absolute rescaling (− 600 to 1300 HU) was applied. Pixel values between the upper and lower limits were resampled at 64 levels uniformly. The morphology, histogram, and texture features were calculated from each VOI on original and filtered images (Laplacian of Gaussian, Wavelet, Square, Square Root, Logarithm, Exponential, Gradient, local binary pattern in 2D, and local binary pattern in 3D). A total of 1874 features were thus extracted.

Several processes were performed to construct a predictive model for STAS using R version 3.5.1 (R Core Team (2018). R: A language and environment for statistical computing. R Foundation for Statistical Computing, Vienna, Austria. URL http://www.R-project.org/). First, a t-test was used to select the features that differed significantly between cases with and without STAS. Second, feature selection with recursive feature elimination was performed in five-fold cross-validation. We tuned the hyperparameters in a training set divided so that the STAS prevalence was equivalent to a test set and assessed how well the model could be generalized to unseen data in five-fold cross validation. The model was built with XGBoost, and the probability of STAS in each patient within a test set was calculated. This method was performed five times for mutually different test sets, and the probability was calculated for all patients.

### Statistical analyses

For the clinical characteristics, we constructed summary statistics and compared the patient characteristics using Fisher’s exact test and chi-square test for categorical outcomes and t-tests or Wilcoxon’s rank sum test for continuous variables, as appropriate.

**Figure 1:**
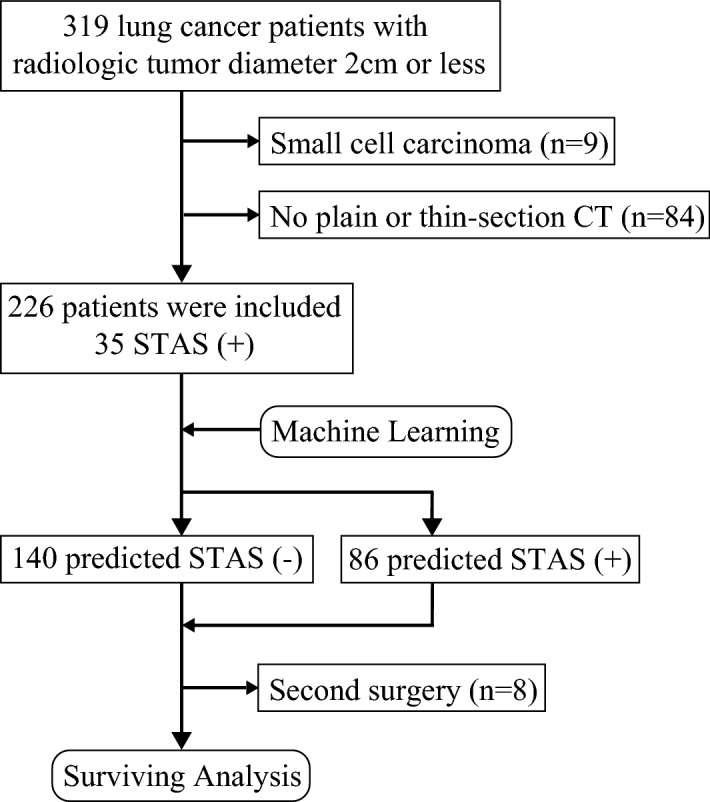
Study cohort flow chart. Peripheral tumors ≤ 2 cm in size on computed tomography that were potential candidates for sublobar resection were enrolled. Four of the patients included in the analysis predicting STAS underwent surgery 2 times during the period. Therefore, the survival curves were analyzed excluding those patients.

Four of the patients included in the analysis predicting STAS underwent surgery 2 times during the study period. Therefore, survival curves were analyzed excluding those patients (Fig. 1). The survival curves were generated with the Kaplan–Meier method and compared with the log-rank test for STAS. The cumulative incidence of recurrence (CIR) was analyzed using a competing risk analysis with Gray’s test^28^. Competing risks are defined as events that prevent the outcome of interest from occurring. Censoring patients at the time of death would lead to a biased probability of the outcome of interest as estimated by the Kaplan–Meier method and would likely overestimate the probability of recurrence. Therefore, the patients were censored if they were alive without recurrence at the latest follow-up.

**Figure 2:**
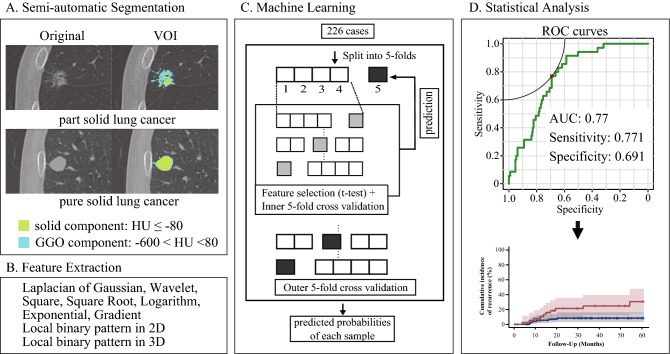
The VOI of lung cancer was delineated, and imaging features were extracted. First, the t-test was used to select features between cases STAS(+)/(−). Second, feature selection with recursive feature elimination was performed. Third, the features were put into XGBoost. The cut-off value was calculated with a ROC analysis, and then the survival curves were analyzed. (**D**, R version 3.5.1, R Core Team (2018). R: A language and environment for statistical computing. R Foundation for Statistical Computing, Vienna, Austria. URL http://www.R-project.org/).

The machine-learning model produced probabilities for STAS that were used for the receiver operating characteristic (ROC) analysis. A threshold to binarize the model output was calculated from the point closest to the top-left part of the ROC curve. Whether the predicted STAS was positive or negative was defined using this threshold. The overall survival (OS) and CIR based on the predicted presence of STAS were analyzed (Fig. 2).

All statistical tests used a 5% level of statistical significance. Statistical analyses were performed with EZR (Saitama Medical Center, Jichi Medical University, Saitama, Japan), which is a graphical user interface for R statistical program (The R Foundation for statistical Computing, Vienna, Austria).

The R codes of machine-learning used in this study have been uploaded to the following URL: https://github.com/hy-423/R_STAS/blob/main/R_result20200902.R.

### Study approval

The study is based on the principles of the Helsinki declaration. The institutional review board (No.3809) at Chiba University Graduate School of Medicine reviewed and approved this study. The need for informed consent was waived by the institutional review board at Chiba University Graduate School of Medicine (No.3809) due to the retrospective nature of the study.

## Results

### Patients’ characteristics

Three hundred and nineteen patients underwent thoracotomy for lung cancer ≤ 2 cm during this study period. Of them, 226 patients ultimately met the study criteria, and 35 (15.4%) patients harbored the STAS histology.

The characteristics of the patients are summarized in Table 1. No significant differences were observed between the STAS (+) and STAS (−) groups with respect to the age, gender, or comorbidities. Regarding the chest CT findings, there was no significant difference in the tumor diameter between the groups. Regarding the consolidation/tumor ratio (C/T ratio), the STAS (+) group included a significantly larger population with a ratio > 0.25 (*p* = 0.03). The surgical procedures (lobectomy or sublobar resection) were equally distributed between the groups. Regarding the pathological findings, nodal metastasis was found in 9 patients (26%) (*p* < 0.001) in the STAS (+) group; the overall p-Stage was thus significantly higher in the STAS (+) group than in the STAS (−) group. Lymphatic invasion was more frequently observed in the STAS (+) group than in the STAS (−) group (*p* < 0.01). There was no significant difference in the incidence of an EGFR mutation between the groups (*p* = 0.70).

**Table 1:** Patients’ characteristics.

Total (N = 226)	STAS negative (n = 191)	STAS positive (n = 35)	*p* value
Age (y) (median, range)	70 (39–88)	68 (38–89)	0.29
Gender, male/female	103 (54%)/88 (46%)	20 (57%)/15 (43%)	0.85
Brinckmann index (median, range)	300 (0–2760)	500 (0–1600)	0.33
FEV1 (L)	2.13 (0.95–4.11)	2.08 (0.9–3.95)	0.33
FEV1 predicted (%)	90.6 (45.9–140.5)	90.2 (49.7–138.7)	0.81
**Comorbidities**			
Chronic obstructive pulmonary disease	69 (36%)	14 (40%)	0.71
Cardiac disorder	36 (19%)	9 (26%)	0.36
Hypertension	62 (32%)	10 (29%)	0.70
Diabetes	33 (17%)	3 (9%)	0.31
Radiologic tumor diameter	14.0 (5.6–2.0)	14.2 (7.0–19.0)	0.82
C/T ratio ≦0.25/ > 0.25	40 (21%)/151 (79%)	2 (6%)/33 (94%)	0.03
**Surgical procedure**			0.28
Lobectomy	86 (45%)	20 (57%)	
Segmentectomy	70 (37%)	8 (23%)	
Wedge resection	35 (18%)	7 (20%)	
**Tumor histology**			< 0.01
Adenocarcinoma	150 (79%)	31 (89%)	
AIS, MIA/Lepidic/Papillary/Acinar/Solid/Micropapillary/Variant	68 (45%)/12 (8%)/46 (31%)/10 (7%)/ 5 (3%)/0 (0%)/9 (6%)	1 (3%)/0 (0%)/18 (58%)/4 (13%)/ 0 (0%)/4 (13%)/4 (13%)	< 0.001
Squamous cell carcinoma	34 (18%)	1 (3%)	
Others	7 (4%)	3 (9%)	
**Pathologic stage**			
T1a/T1b/T2/T3/T4	148 (77%)/11 (6%)/30 (16%)/2 (1%)/0(0%)	22 (63%)/7 (20%)/6 (17%)/0 (0%)/0 (0%)	0.07
N0/N1/N2/N3/NX	152 (79%)/1 (1%)/5 (3%)/0 (0%)/33 (17%)	24 (69%)/5 (14%)/4 (11%)/0 (0%)/2 (6%)	< 0.001
Stage IA/IB/II/III/IV	156 (81%)/26 (14%)/4 (2%)/5 (3%)/0 (0%)	22 (63%)/4 (11%)/5 (15%)/4 (11%)/0 (0%)	< 0.01
Pathologic lymphatic invasion	12 (6%)	8 (23%)	< 0.01
Pathologic vascular invasion	34 (18%)	11 (31%)	0.07
Pathologic plural invasion	30 (16%)	5 (14%)	1.00
EGFR Wildtype/Mutant	8 (47%)/9 (53%)	4 (40%)/6 (60%)	1.00

### The CIR and OS

**Figure 3:**
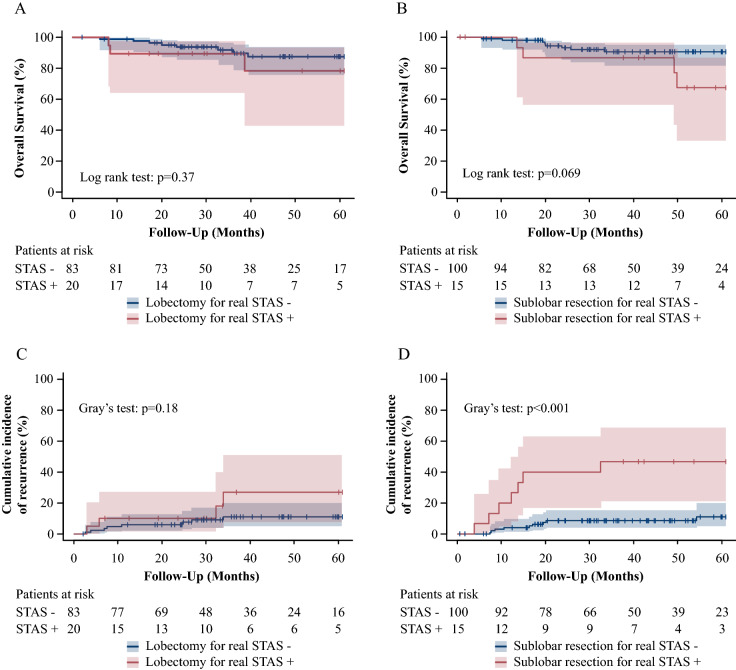
(R version 3.5.1, R Core Team (2018). R: A language and environment for statistical computing. R Foundation for Statistical Computing, Vienna, Austria. URL http://www.R-project.org/). The OS and CIR associated with real STAS in lobectomy and sublobar resection. Shaded areas represent 95% confidence limits. (**A**) The OS in patients who underwent lobectomy. (**B**) The OS in patients who underwent sublobar resection. (**C**) The CIR in patients who underwent lobectomy. (**D**) The CIR in patients who underwent sublobar resetion.

**Figure 4:**
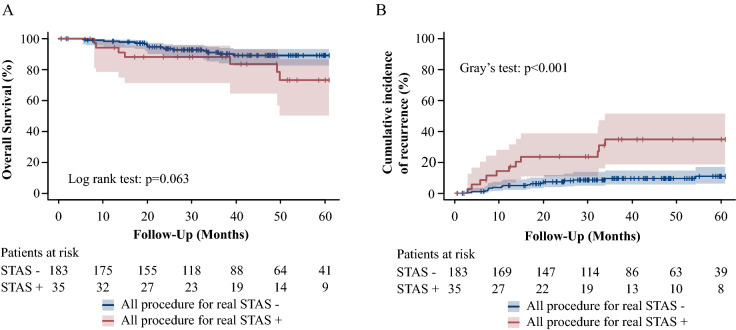
(R version 3.5.1, R Core Team (2018). R: A language and environment for statistical computing. R Foundation for Statistical Computing, Vienna, Austria. URL http://www.R-project.org/). The OS and CIR associated with real STAS in all procedure. Shaded areas represent 95% confidence limits. (**A**) The OS in all patients. (**B**) The CIR in all patients.

The median observation period was 36.9 months (interquartile range 24.4–60.4 months). The CIR was compared between the STAS (+) and STAS (−) groups and while there was no significant difference in the CIR among patients who underwent lobectomy (*p* = 0.18), the STAS (+) group showed a higher CIR after sublobar resection than the STAS (−) group (*p* < 0.001) (Fig. 3C,D). There was no significant difference in the OS between the STAS (+) and (−) groups among lobectomy (*p* = 0.37) or sublobar resection (*p* = 0.069) patients (Fig. 3A,B). For all procedures, the STAS (+) group showed a higher CIR (*p* < 0.001) than the STAS (−) group but no significant difference in the OS (*p* = 0.063) (Fig. 4A,B).

### The CIR and OS based on STAS predicted by radiomics

**Figure 5:**
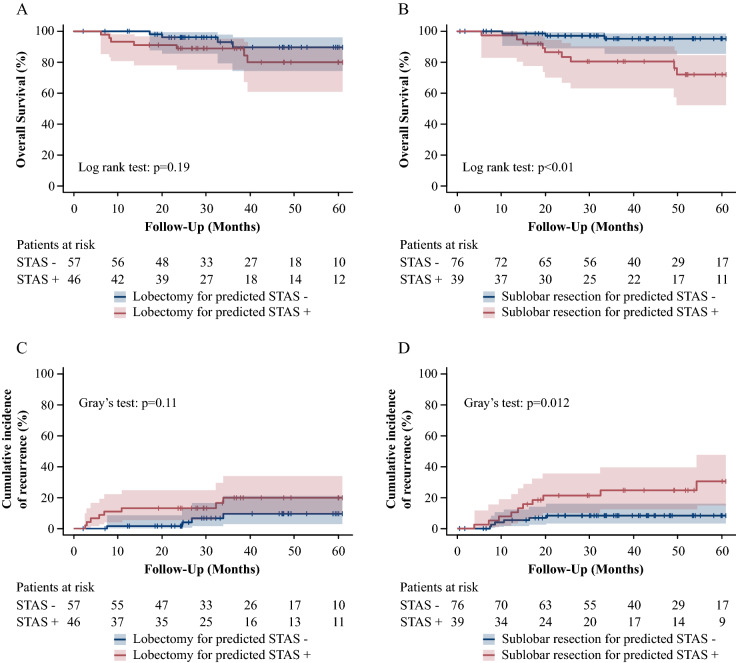
(R version 3.5.1, R Core Team (2018). R: A language and environment for statistical computing. R Foundation for Statistical Computing, Vienna, Austria. URL http://www.R-project.org/). Survival curves based on the model predicted by radiomics. The OS and CIR associated with predicted-STAS in lobectomy and sublobar resection. Shaded areas represent 95% confidence limits. (**A**) The OS in patients who underwent lobectomy. (**B**) The OS in patients who underwent sublobar resection. (**C**) The CIR in patients who underwent lobectomy. (**D**)The CIR in patients who underwent sublobar resection.

**Figure 6:**
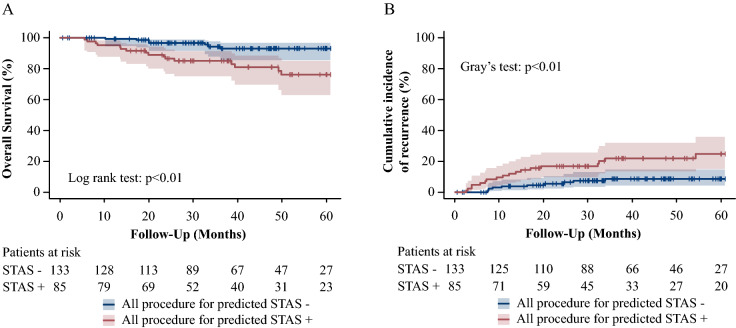
(R version 3.5.1, R Core Team (2018). R: A language and environment for statistical computing. R Foundation for Statistical Computing, Vienna, Austria. URL http://www.R-project.org/). Survival curves based on the model predicted by radiomics in all procedure. Shaded areas represent 95% confidence limits. (**A**) The OS in all patients. (**B**) The CIR in all patients.

The AUC for the positivity of STAS was 0.77. The likelihood of STAS was determined based on the radiomics, and patients were then divided into predicted-STAS (+) and (−) groups. For the patients who underwent lobectomy, there was no significant difference in the CIR (*p* = 0.11) or OS (*p* = 0.19) (Fig. 5A,C) between the groups. However, for patients who underwent sublobar resection, the predicted-STAS (+) group showed a higher incidence of CIR (*p* = 0.012) and poorer OS (*p* < 0.01) than the predicted-STAS (−) group (Fig. 5B,D). For all procedures, the predicted-STAS (+) group showed a higher CIR and OS than the predicted-STAS (−) group (*p* < 0.01) (Fig. 6A,B).

## Discussion

Radiomics, the process of converting radiographic images into quantifiable information, helps develop prediction models that can potentially improve the accuracy of a diagnosis^29^. In a previous radiomics study reported by Jiang et al.^21^, their predicted model was built using the Random Forest algorithm with training (n = 195) and validation cohorts (n = 84) for 107 radiologic features. Their model was able to effectively predict STAS-positive tumors with an AUC of 0.7. In another study, Chen et al.^22^ constructed a prediction model using 88 features extracted from stage I adenocarcinomas using the Naïve Bayes algorithm, which also effectively predicted STAS positive tumors with an AUC of 0.63 for a training cohort of 233 cases and 0.69 for a validation cohort of 112 cases. In the present study, the prediction model was constructed by XGBoost for 1874 features extracted from 226 peripherally located small NSCLC and was able to predict the presence of STAS with an AUC of 0.77.

Our study focused on two clinical questions. The first one was whether or not a preoperative diagnosis and surgery procedure selection were possible using a model built by machine-learning-based radiomics. The AUC of 0.77 is regarded as moderate accuracy and is better than that obtained in previous studies; however, this accuracy will need to be improved for clinical use. The second one was whether or not the model was useful for determining the ideal type of surgery. STAS (micropapillary pattern)-positive NSCLC, especially adenocarcinoma, is reportedly associated with recurrence after segmentectomy but not after lobectomy^30^. Therefore, the present study focused on peripherally located small (≤ 2 cm) lesions. Our subjects also included non-adenocarcinoma patients, since they sometimes have STAS. In total, 4 (8.9%) of the 45 non-adenocarcinoma tumors possessed STAS in our study. This second question was also positively answered based on the results of a survival analysis for the predicted-STAS (+) group, who showed a higher incidence of recurrence and a worse survival than the predicted-STAS (−) group when sublobar resection was performed.

The invasive morphology of STAS, characterized by micropapillary clusters, solid nests or single cells beyond the edge of the tumor extending into the air spaces in the surrounding lung parenchyma^2^, resulted in poor clinical outcomes, even for early-stage disease, in patients with NSCLC^3,4^. Kadota et al. described an increased risk of developing CIR in STAS-positive pulmonary adenocarcinomas treated with sublobar resection; however, this risk was mitigated by lobectomy^31^. The preoperative and intraoperative diagnoses of STAS therefore seem important for deciding on the surgical plan (sublobar resection or lobectomy) for peripheral small-sized lung cancer. However, the detection of STAS remains challenging prior to thoracotomy by preoperative radiology and cytology, as well as by frozen section analyses during surgery. The sensitivity of diagnosing STAS by airway secretion cytology^32^ or frozen sections during surgery^33^ is not satisfactory for clinical use. STAS-positive pulmonary adenocarcinoma^34^ is frequently observed as solid nodules on computed tomography with a low incidence of ground-glass attenuation, showing abnormal avidity on PET^35,36^. We select lobectomy rather than sublobar resection for lesions deemed suspicious on radiology^37^. However, the sensitivity and specificity of conventional CT feature interpretation by radiologists were not satisfactory for predicting STAS. A few reports^21^^,^^22,38,39^ have described the utility of radiomics for predicting STAS based on the findings of radiological examinations.

Machine-learning-based radiomics are a powerful tool in thoracic oncology, using an exploratory analysis to detect complex patterns that cannot be recognized by traditional analyses. In particular, some publications reported that quantifiable radiomics features extracted from the VOI within CT images can provide more information not only for subtype but also genetic information and immune infiltration of tumors^18–20^. Most cancer cases are subjected to multiple rounds of CT, MRI, and PET-CT prior to thoracotomy, accumulating a large amount of data; however, modern CT, MRI and combined PET-CT units are not standardized for image acquisition and reconstruction protocols. With simple machine-learning of radiological images, overfitting cannot be completely avoided, resulting in some input data bias, with the results affected not only by the biological features, but also by the imaging protocols. In the present study, we first extracted the image feature values and then applied the data to machine-learning algorithms. To avoid showing results that overfit the partitioning of a particular data set, the cross-validated models were evaluated in a test set five times using different sets each time.

Several limitations associated with the present study warrant mention. First, our study was a retrospective analysis of CT findings based on the results of pathology, not a prospective study. Because the ground truth diagnosis associated with medical imaging data analyzes the accumulated data, it necessarily focuses on the retrospective aspects of such studies. A prospective study in which surgical procedures are controlled by the result of radiomics prediction may be warranted in the future. Second, STAS has not been defined for many years, and even pathologists may have difficulty diagnosing this entity. No clear criteria for distinguishing STAS from artifacts have been established^26^. We re-evaluated each diagnosis to reduce the diagnostic error. The postoperative outcomes were similar to those of previous reports, which might have ensured the quality of the STAS diagnosis. Third, this analysis included some cases of high-grade neuroendocrine tumors, such as combined small-cell lung cancer and large-cell neuroendocrine carcinoma, which were diagnosed by a final pathological evaluation. We focused on the utility of the pre-operative prediction of STAS-positive lung cancer using radiomics-based technology; these cases were therefore not excluded. Fourth, we did not set a validation cohort to confirm the prediction model, although five-times-repeated five-fold cross-validation was applied. However, the survival data clearly demonstrated the clinical usefulness of the prediction model. In our study, the presence of STAS was a strong predictor of surgical outcomes that was not restricted to a histologic type. The further accumulation of cases with a prospective cohort study should be conducted in the future.

In conclusion, radiomics-based analyses utilizing chest CT imaging findings were found to be feasible for predicting STAS as well as the postoperative outcomes. The survival curves suggested if the STAS could be predicted preoperatively by radiomics-based analyses, then we could select lobectomy to ensure better outcomes. This technology would greatly aid surgeons in selecting the optimal surgical treatment plan and it is also important for lung cancer clinics treating small nodules detected by CT screening.
